# Image-Based Identification and Localisation of Changes in Intraoperative Brain Tumour Resection

**DOI:** 10.1007/s10278-025-01744-8

**Published:** 2025-11-07

**Authors:** Adil Jahouh, Andrei Jalba, Maxime Chamberland

**Affiliations:** Department of Mathematics and Computer Science, Eindhoven University of Technology, Eindhoven, Netherlands

**Keywords:** Siamese network, Intraoperative, Resection, Tumour, Change, Segmentation

## Abstract

Conventional brain tumour progression tracking methods inherently assume consistent image acquisition conditions between baseline and follow-up scans. In practice, however, MRI data often suffer from harmonisation issues due to variations in acquisition protocols, scanner settings, or patient positioning. To address this, we propose an image-based approach capable of identifying and localising changes between baseline and follow-up MRI scans without requiring independent registration or extensive pre-processing. Our registration-invariant method leverages the internal feature maps of a Siamese network composed of two segmentation models based on dilated convolutions, allowing it to learn long-context spatial features. These features are used to detect and localise changes directly from the input images, which are then enhanced using panchromatic sharpening to emphasise both high-level structural and pixel-level differences within the resulting change map. When tested on previously unseen, unregistered MRI scans, the method outperformed baseline models in identifying and localising resected tumour tissue ($\boldsymbol{F}_{\boldsymbol{1}} \boldsymbol{= 0.64}$ and $\boldsymbol{IoU = 0.57}$ versus baseline $\boldsymbol{F}_{\boldsymbol{1}} \boldsymbol{= 0.11}$ and $\boldsymbol{IoU = 0.07}$). This performance remained robust under conditions of augmentation, noise, and misalignment ($\boldsymbol{F}_{\boldsymbol{1}} \boldsymbol{= 0.55}$ and $\boldsymbol{IoU = 0.45}$), highlighting its resilience to real-world imaging variability. Overall, the proposed approach demonstrates strong robustness to spatial misalignment and offers a promising solution for tracking tumour progression and regression in clinical environments where reliable registration cannot be guaranteed.

## Introduction

The precise detection of changes in longitudinal imaging plays a critical role in various fields, including the automotive industry for advancing self-driving vehicles, the medical field for monitoring patient progression, and the agriculture industry for optimising crop management. This concept of change detection (CD) was originally referred to as the process of identifying differences in the state of an object or phenomenon by observing it at different times [31]. CD in many contexts is often extended and involves not only determining whether a change has occurred (identification), but also the localisation of the change, addressing exactly what has changed and where it has occurred [26]. CD between time points in conventional approaches, such as difference or dissimilarity maps (collectively referred to as DM) [30], often rely on pre-processed, aligned, and normalised images to identify these dissimilarities in a common field. This becomes problematic when the images are not aligned [1], taken from angles which cannot be aligned or incompatible due to differing acquisition protocols or perspectives, making it difficult to construct dissimilarity maps without extensive post-processing.

In the medical and more specifically neurosurgical context, this challenge is of crucial importance. Surgeons often compare preoperative and intraoperative scans to evaluate the extent of tumour resection or oppositely disease progression. However, intraoperative imaging presents significant difficulties for such comparisons, as the brain undergoes structural shifts during surgery (brain shift). Furthermore, the issue of deformation and resection cavities makes it nearly impossible to align intraoperative scans accurately with preoperative ones. These changes complicate direct voxel-to-voxel comparisons and hinder reliable assessment of residual tumour tissue.

A common way to perform disease progression tracking is by using a DM [2, 21, 29]. This approach requires all scans to be aligned and standardised to account for variations. Pre-processing removes non-brain structures, aligns scans using rigid or affine registration, and normalises intensity variations with histogram matching. After alignment and harmonisation, changes are detected by subtracting the intensity values between scans. Lastly, some sort of post-processing is applied to reduce false positive clusters of voxels. This approach is therefore tied to careful pre-processing by registration, artefact removal, and normalisation.

Another common strategy for detecting change is anomaly detection (AD) [5]. AD is, however, not sufficient for applications where localisation of changes is essential. Therefore, many anomaly detection models can integrate anomaly localisation (AL) [33] by using their reconstruction error, generative properties, or model feature descriptors to highlight regions of anomalous behaviour. Reconstruction-based approaches detect anomalies by measuring the reconstruction error when a model reconstructs a data point [4, 8, 9]. An example of this is the use of autoencoders (AEs) [4] to detect anomalous white matter bundles within dMRI data. Generative methods, on the other hand, model a latent space trained on normative data and detect anomalies by deviations in this space [19, 27, 33]. AEs can be extended into variational autoencoders (VAEs), which model a full distribution and allow controlled sampling [18]. While AL methods can be extended to CD [14], their effectiveness is very dependent on their training data as they are not inherently trained on changes but on out-of-distribution (OOD) verifications and normative data.

DM and AL methods are sensitive to translation and noise, limiting their performance on unregistered image pairs. Scene change detection addresses this change detection task in scenarios involving viewpoint variations, illumination, and other types of noise by leveraging similarity models such as Siamese networks that are trained to distinguish true scene changes from those induced by such variations [10, 13]. These models typically incorporate contrastive loss functions and are designed to penalise the model only for meaningful differences, allowing them to maintain robustness in the presence of challenging visual discrepancies [13].

More recently, methods involving deep neural networks have significantly advanced CD. Deep convolutional neural networks (Deep CNNs) are capable of learning complex features by leveraging the hierarchical structure of the network, allowing it to capture important relationships between pixels within an image. Moreover, CNNs can effectively handle noise by incorporating translation invariance into the model through augmentation and dilation, enabling robust detection in both classification and CD tasks. This, in combination with Siamese networks which allow for multiple inputs to be compared, allows for a novel CD method that not only classifies if a pair or multiple images have changed, but also localises and identifies the changes.

In this work, we explore a registration-invariant approach (RiA) to CD, inspired by Guo et al. [13]. Our method is designed to identify, localise, and visualise changes in intraoperative brain tumour resection using a deep learning model. We propose a method that leverages the feature maps within Siamese CNNs to identify and localise changes between a baseline and follow-up image without the need for registration (the spatial alignment in a shared coordinate space) or pre-processing. These changes are then evaluated with an area-based evaluation metric using the ground truth of the resected or otherwise changed tissue and applied to the follow-up image using panchromatic sharpening to highlight high-level features and pixel-level changes within the change map. Although our present study analyses preoperative and intraoperative scans offline, the proposed framework can be deployed in near-real-time once trained, enabling intraoperative feedback during tumour resection.

## Materials and Methods

### Dataset

The dataset used in this research is provided by the ReMIND database [16], which contains data from 114 brain tumour patients who underwent tumour resection. The dataset includes patients diagnosed with gliomas (*n*=92), metastases (*n*=11), and other tumour types (*n*=11). Two primary imaging modalities were used for brain scans: MRI (preoperative and intraoperative) and ultrasound (intraoperative). However, for the brain scans used in this study, only MRI data were included. Preoperative MRIs were acquired at multiple institutions using different scanners and heterogeneous acquisition protocols, with Siemens being the most common manufacturer. Detailed acquisition parameters and protocol descriptions are provided in [16]. In contrast, all intraoperative MRIs were obtained at Brigham and Women’s Hospital using a standardised protocol on a 3T Siemens Magnetom Verio scanner. For preoperative and intraoperative MRI, multiple structural sequences were acquired, including T1-weighted (T1) post-contrast T1 (ceT1), T2-weighted (T2), and T2-FLAIR. Of these sequences, only T1 and ceT1 were used in this study due to consistent data availability between the preoperative and intraoperative acquisitions.

To ensure the reliability of manual tumour segmentations, all annotations were created by a neurosurgical fellow using BrainLab [12]. These annotations were reviewed and potentially refined, when necessary, by an attending neurosurgeon. As part of the public release of the ReMIND dataset, interobserver variability was quantitatively assessed on a subset of cases, yielding high agreement between independent annotators (Dice scores of 92.2% for enhancing tumours and 86.4% for non-enhancing tumours) [16]. This validation demonstrates that the manual annotations were consistent and not subject to systematic over- or under-segmentation relative to algorithmic performance. Preoperative tumour segmentations were manually performed exclusively on contrast-enhanced T1-weighted MRI, delineating the enhancing tumour region. Intraoperative residual tumour segmentations were manually annotated based on intraoperative MRI (iMRI) to assess any remaining tumour tissue after resection. Due to the limited number of patients with residual tumour masks, segmentations derived from different iMRI structural sequences (e.g. T2-weighted MRI or T2-FLAIR) were also included for the tumour masks used within this study. In contrast to the brain scans, which were restricted to matching preoperative and intraoperative structural sequences, these masks were treated purely as binary delineations of tumour presence rather than as intensity-based imaging data. The final cohort, comprising patients with all required data (including preoperative tumour and residual tumour information), consists of *n* = 65 patients.

The volumetric preoperative and intraoperative brain scans, along with their corresponding tumour segmentations, are stripped of bone and other non-brain tissue, harmonised to a uniform resolution of $256 \times 256 \times 256$, and pre-processed using various tools [11, 22, 32]. Following this, the data are sliced along the sagittal, axial, and coronal planes [25], which generates 768 slices in total across 65 ReMIND patients. Each resulting data point is a 2D array of size 256×256, where a pixel at location (i,j) corresponds to the intensity value at row i and column j, with $i, j \in \{1, \ldots , 256\}$. The intensity values are normalised to the range [0,1], and each slice corresponds to either the preoperative brain ($P_0$), intraoperative brain ($P_1$), preoperative tumour ($T_0$), or residual tumour ($T_1$).

Using these slices, we generate a ground truth map for the resected tissue, denoted as ΔT, by retaining only the tumour regions present in $T_0$ that are no longer present in $T_1$. This is achieved via pixel-wise masking. Each value in ΔT represents a binary label indicating either resected (0) or unchanged (1) tissue.

### Baseline

For benchmarking, we firstly construct baseline methods inspired by Lesjak et al. [21]. This methodology allows for an interpretable and conventional method for longitudinal change detection between brain scans. It represents a “best-case scenario” for traditional methods, as the baseline benefits from fully aligned and registered images, ensuring that any differences in performance reflect the model’s capability rather than pre-processing quality. In these methods, both the preoperative and intraoperative images are first normalised and registered to ensure valuable results. After pre-processing the slice data, the dissimilarity map (${D}_{baseline}$) is computed by taking the absolute pixel-wise difference between the intraoperative image ($P_{\text {1}}$) and the preoperative image ($P_{\text {0}}$).

**Fig. 1:**
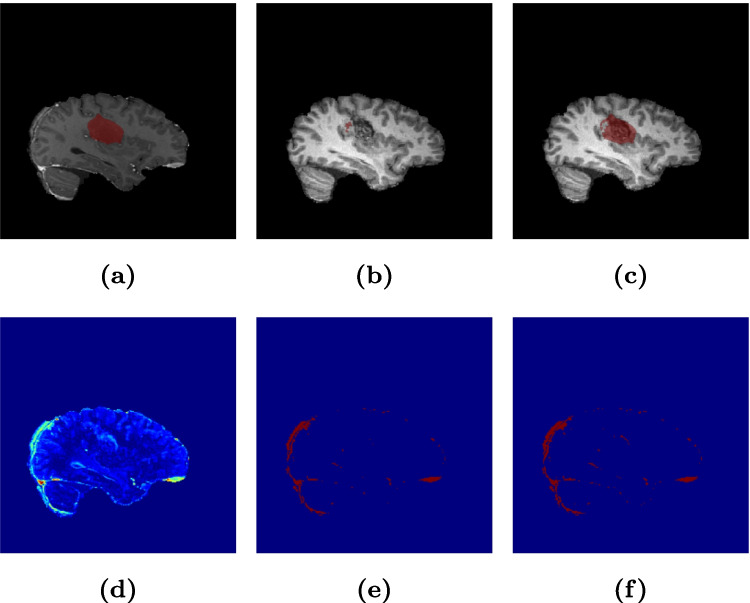
Sample baseline results of ReMIND patient 39, showing a sagittal mid-point slice. **a** $T_0$ superimposed on $P_0$. **b** $T_1$ superimposed onto $P_1$. **c** ΔT superimposed onto $P_1$. **d** Raw difference map $D_{\text {baseline}}$. **e** Fixed threshold change map $\Delta \hat{T}_{\text {thresh}}$ for γ=0.3. **f** *Z*-score change map $\Delta \hat{T}_{z\text {-score}}$

**Fig. 2:**
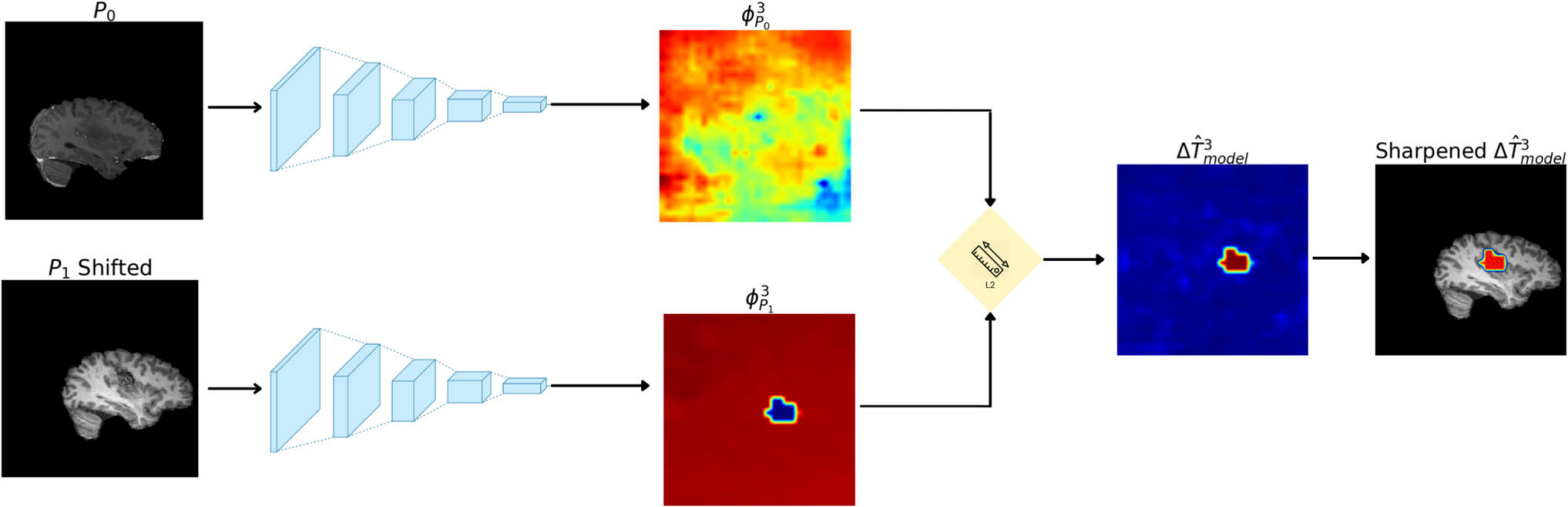
A simplified overview of the inference process of a trained registration-invariant approach. The network accepts two images ($P_0, P_1$) as input and sequentially applies convolutional and pooling layers to extract hierarchical spatial feature representations ($\phi ^3_{P_0}, \phi ^3_{P_1}$) in the form of feature maps at the final layer (l=3). These feature maps are then compared using the L2 norm to construct a change map ($\Delta \hat{T}^3_{model}$), which is sharpened using high-level features of $P_1$ and superimposed onto $P_1$

It is important to note that $D_{baseline}$ is not the final prediction of the change; rather, it represents a difference map that highlights raw changes between the two images. To obtain a final prediction map, a post-processing step is required. In this step, a *z*-score threshold as described in Eq. 1 is applied to the difference map. This way, only pixels with intensity values deviating by more than four standard deviations (σ) from the mean (μ) of the overall normalised image intensity are retained.

1 $$\begin{aligned} Z_{(x,y)}=\frac{D_{baseline(x,y)}-\mu }{\sigma } \end{aligned}$$

Furthermore, as the distribution of the data is heavily skewed towards zero intensities due to the large background region, a thresholding technique based on fixed thresholds ($\gamma \in \{0.1, 0.3, 0.5\}$) over $D_{baseline}$ is also applied for the alternative difference map approach. The resulting filtered change maps, $\Delta \hat{T}_{z-score}$ and $\Delta \hat{T}_{thresh}$, constitute the final predictions of the tumour region by the baseline method, as can be seen in Fig. 1f and e.

### Registration-Invariant Approach

The registration and harmonisation are, however, not required for our approach. This registration was solely performed to generate a DM (${D}_{baseline}$) and to label each slice as the dataset is moved into a 2-dimensional space. Once the dataset is converted, the data is then augmented by shifting and rotation to simulate partial slices and unregistered pairs. This way allows us to test the robustness of the model, adding an extra constraint to its evaluation as opposed to the baseline methods.

One model architecture, known for its ability to discern between anomalies, is the Siamese network. It does this by accepting two inputs and passing the inputs to each subnetwork. These two subnetworks, however, share the same weights and thus technically pass each input to the same network. The Siamese network, however, is not a model, but more so a network architecture. Supplying a structural framework to place models in a way that pairs or higher-order combinations of inputs can be compared. To implement this design pattern, the Siamese network expects a model to be used as its subnetwork. CD has commonly performed with a segmentation-based model [10, 15]. One model, commonly known for its accurate long-range segmentation capabilities, is DeepLab [6, 7]. Using its dilated convolutions at different dilation rates and stacking these, it is able to combine multi-scale features while retaining its spatial resolution. This way, it can segment fine details in long-range contexts. Furthermore, it is able to exploit even wider feature ranges by implementing global features using global average pooling before the stacking procedure [24].

**Table 1:** Model hyperparameters

Parameter	Value/range	Description
Learning rate (lr)	0.001	Adam optimizer learning rate
Epochs	200	Maximum number of training epochs
Patience	8	Early stopping criteria
Batch size	16	Training/validation/test batch size
Optimizer type	Adam	Optimizer used
Loss type	TCL	Thresholded Contrastive Loss
Margin range	0–6	Margin for dissimilar pairs in contrastive loss
Distance flag	Euclidean	Distance metric (Euclidean, Manhattan, Cosine)
Threshold range	0.0–0.9	Threshold parameter in TCL loss
Dropout	0.5	Standard dropout probability in various layers
Dilation rates	2, 3, 6, 9, 12, 18	Dilation rates for atrous convolutions
Normalisation scale	1.0	Scale factor for feature normalisation
Max shift *X*	50 pixels	Maximum horizontal shift for augmentation
Max shift *Y*	50 pixels	Maximum vertical shift for augmentation
Tumour sensitivity	0.30	Threshold for tumour pixel sensitivity
Segmentation value index	0.30	Threshold for tumour segmentation evaluation
Train/Val/test	80%/10%/10%	Dataset split ratios

**Fig. 3:**
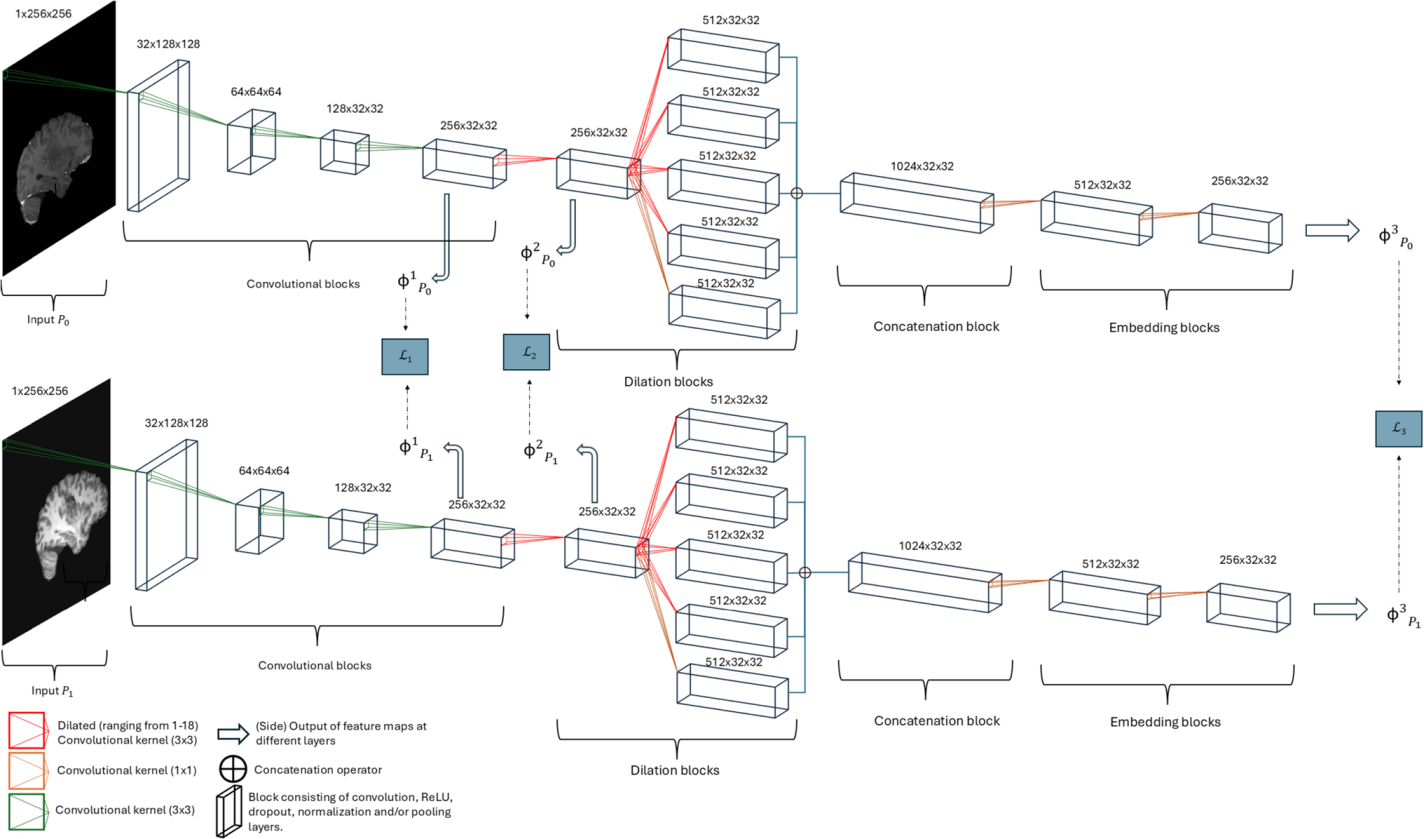
An in-depth overview of the training process within the Siamese network architecture

In our approach (see Fig. 2), each branch of the Siamese network is implemented using a segmentation model with various dilation rates and global pooling as its feature extraction backbone. Given two input images, such as $P_0$ and $P_1$, each is processed independently through a segmentation subnetwork, which returns a change map based on the pixel-wise L2 norm between the feature maps. Using these distances, we are able to localise the exact changes by using the pixel-wise difference at the final output layer ($\Delta \hat{T}^3_{model}$) and generate a binary change label ($\hat{y}$) by performing a global distance measure at the final output layer. This would in turn give us a clear global change prediction and a change detection map. We then evaluate our model according to multiple statistics, both pixel-wise and globally, to quantify our model performance. Lastly, we apply a technique called panchromatic sharpening to visually evaluate structural changes to white or grey matter.

#### Training Strategy

We trained the model using the hyperparameters listed in Table 1. Training was conducted in an environment equipped with 10 CPU cores, 75 GB RAM, and dual RTX 2080 Ti GPUs, on which the model converged within approximately two hours. During training, our model extracts a high-dimensional feature representation (ϕ) for each input at three different stages of the model, as shown in Fig. 3. Each feature map extracted in an output layer *l* ($l \in {1, 2, 3}$) for input $P_i$, with $i \in {0, 1}$, is denoted as $\phi _{P_i}^l$.

Due to the model’s early down-sampling by max-pooling layers, the dimensions of each feature map at all output layers *l* are 256 × 32 × 32 (channels, height, and width, respectively) as can be seen in Table 2. This, therefore, results in a highly downsampled feature map compared to the original dimensions. To perform any sort of evaluation, ΔT is downsampled to match the dimensions of $\phi _{P_i}^l$, using bilinear interpolation, before being processed.

**Table 2:** Siamese network (single branch) parameter summary

Block	Description	Output shape	Parameters
Input	Grayscale image	$1 \times 256 \times 256$	–
conv1	Initial feature extraction	$32 \times 128 \times 128$	9504
conv2	Feature extraction	$64 \times 64 \times 64$	55,296
conv3	Feature extraction	$128 \times 32 \times 32$	368,640
conv4	Feature extraction	$256 \times 32 \times 32$	1,474,560
conv5	Dilated convolution	$256 \times 32 \times 32$	1,769,472
aspp_conv1	ASPP branch 1 (1×1 conv)	$512 \times 32 \times 32$	131,584
aspp_conv2	ASPP branch 2 (3×3, dilation=6)	$512 \times 32 \times 32$	1,180,160
aspp_conv3	ASPP branch 3 (3×3, dilation=12)	$512 \times 32 \times 32$	1,180,160
aspp_conv4	ASPP branch 4 (3×3, dilation=18)	$512 \times 32 \times 32$	1,180,160
aspp_pool	ASPP global pooling	$512 \times 32 \times 32$	131,584
aspp_final	ASPP fusion	$512 \times 32 \times 32$	1,311,232
embedding_layer	Final embedding	$256 \times 32 \times 32$	131,072
Total			8,923,424

Training the model to detect relevant changes of the resected tissue requires a loss function that is able to pixel-wise correct the segmentation based on ΔT. These feature maps ($\phi _{P_i}^l$) are passed, together with a downsampled ΔT, to an adapted loss function as described in Eq. 2. Here, a pixel-wise L2 norm is applied to the feature maps to compute the distance between the two segmentation feature maps, highlighting the changed area. Furthermore, we use two crucial “boundary” parameters, τ and *m*, such that the distance between unchanged pixels ($\Delta T_{(x,y)} = 1$) between the feature maps does not contribute to the loss if their distance is below the value of τ. Similarly, for *m*, changed pixels do not contribute to the loss if their distance exceeds the margin; this way, we not only prevent overfitting and introduce robustness but also take into account noise. This noise can come in the form of misalignment, acquisition differences, intensity variations, etc. We calculate our losses over all these output layers, as shown in Fig. 3. This allows the segmentation model within the Siamese network to control the information during training before further pooling is applied, to help shape feature maps during early layers and balance between low and high-level details, to name a few.

2 $$\begin{aligned} \mathcal {L}_l(\phi _{P_{0}(x,y)}^l, \phi _{P_{1}(x,y)}^l, \Delta T_{(x,y)}) = {\left\{ \begin{array}{ll} \left( \max \left( 0, \Vert (\phi _{P_{0}(x,y)}^l - \phi _{P_{1}(x,y)}^l)\Vert _2 - \tau \right) \right) ^2 \\ \hspace{2em} \text {if } \Delta T_{(x,y)} = 1 \\ \left( \max \left( 0, m - \Vert (\phi _{P_{0}(x,y)}^l - \phi _{P_{1}(x,y)}^l)\Vert _2 \right) \right) ^2 \\ \hspace{2em} \text {if } \Delta T_{(x,y)} = 0 \end{array}\right. } \end{aligned}$$

By leveraging the supervised loss over each output pair ($\phi _{P_0}^l$, $\phi _{P_1}^l$) at layer *l*, the model learns to detect large-scale structural differences as well as fine-grained pixel-wise variations. This ensures robust change localisation across different levels of detail. Following this, the pixel-wise loss is accumulated and averaged before being backpropagated through the model.

#### Panchromatic Sharpening

Using a technique called panchromatic sharpening [20], a lower resolution image such as a $\Delta \hat{T}^3_{model}$ can be fused with the high-resolution panchromatic image, leveraging the high-level features of the panchromatic image and the intensity values of the resected tissue. This way, we are able to highlight the changed region and retain the high-level features of the underlying $P_1$ image.

$P_1$, however, can be an intraoperative image where, due to resection, the tissue is completely removed. This causes the panchromatic sharpening to malfunction due to the matrix-wise multiplication of an empty area in the panchromatic image with intensity values in $\Delta \hat{T}^3_{model}$. This would completely omit a changed area due to the absence of features in the intraoperative. Panchromatic sharpening is therefore applied to the $\Delta \hat{T}^3_{model}$, instead of $P_1$, and then is superimposed on $P_1$. This allows the panchromatic sharpening to take into account high-level features and not be skewed by missing tissue. We apply the following functions to $\Delta \hat{T}^3_{model}$ and $P_1$:

3 $$\begin{aligned} K_{\text {1}} = P_1 \star G_{\sigma } \end{aligned}$$

where $G_{\sigma }$ is a Gaussian kernel and ⋆ denotes a 2D convolution, applied to $P_1$ to create a blurred version of the image called $K_1$. After which, we use this blurred version to extract the high-level features $f_1$ of our follow-up, intraoperative image:

4 $$\begin{aligned} f_1 = P_1 - K_{\text {1}} \end{aligned}$$

We can then apply these high-level features to $\Delta \hat{T}^3_{model}$:

5 $$\begin{aligned} S = \Delta \hat{T}^3_{model} \cdot (1 + \alpha f_1) \end{aligned}$$

where α is a weighting coefficient that controls the influence of high-frequency features $f_1$ on the final sharpened change map *S*. Using this panchromatic sharpened image, we are able to visually detect the exact changes within the brain microstructure. We are also able to calculate finer metrics as the panchromatic sharpening performs an inherent filtering by, at times, nullifying false positives. This should happen whenever the model overshoots changes at coordinates where there is no tissue at all, nullifying these by using pixel-wise multiplication in Eq. 5. We visually assess this sharpened output in comparison to ΔT.

**Table 3:** Baseline performance metrics

Method	$F_1$ score$^{1}$	IoU$^{1}$	Precision$^{1}$	Recall$^{1}$
Fixed (γ=0.1)	0.08	0.04	0.05	**0**.**65**
Fixed (γ=0.3)	**0**.**11**	**0**.**07**	0.10	0.24
Fixed (γ=0.5)	0.06	0.04	**0**.**10**	0.06
*Z*-score	0.11	0.07	0.10	0.26

Performance metrics for different thresholding methods for our baseline methods **performed on registered image pairs**. The best-performing method for each metric is highlighted in bold

$^{1}$Decision threshold used for all metrics is based on the optimal $F_1$ score

## Results

### Baseline

We estimate a difference map by simple pixel-wise subtraction of a registered baseline and follow-up image pair. No augmentation is applied as the baseline methods would not perform well on shifted or rotated images, resulting in an unfair comparison. The difference map is filtered by a *z*-score and a fixed threshold. Using this, we calculate the evaluation metrics for these generated change maps shown in Table 3.

**Fig. 4:**
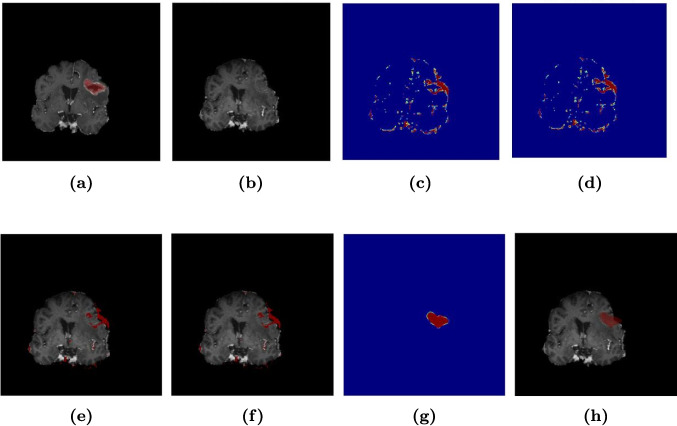
Sample baseline results of ReMIND patient 86, showing a registered coronal mid-point slice. **a** $T_0$ superimposed on $P_0$. **b** $T_1$ superimposed onto $P_1$. **c** Fixed threshold change map $\Delta \hat{T}_{thresh}$ for γ=0.3. **d** *Z*-score change map $\Delta \hat{T}_z\text {-score}$. **e** $\Delta \hat{T}_{thresh}$ superimposed onto $P_1$. **f** $\Delta \hat{T}_{z\text {-score}}$ superimposed onto $P_1$. **g** The ground truth ΔT. **h** The ground truth ΔT superimposed onto $P_1$

**Fig. 5:**
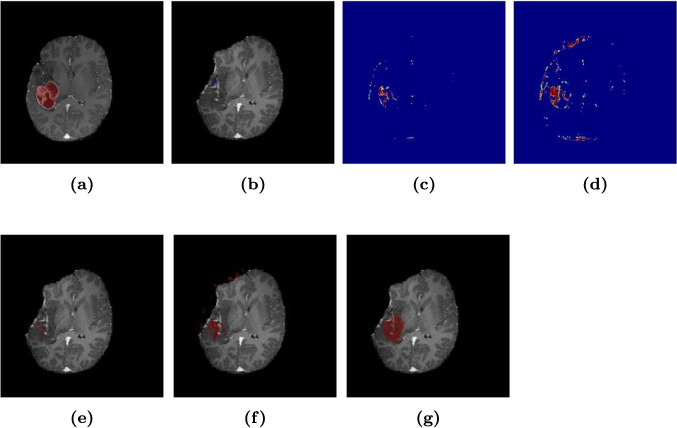
Sample baseline results of ReMIND patient 57, showing a registered axial mid-point slice. **a** $T_0$ superimposed on $P_0$. **b** $T_1$ superimposed onto $P_1$. **c** Fixed threshold change map $\Delta \hat{T}_{thresh}$ for γ=0.5. **d** *Z*-score change map $\Delta \hat{T}_z\text {-score}$. **e** $\Delta \hat{T}_{thresh}$ superimposed onto $P_1$. **f** $\Delta \hat{T}_{z\text {-score}}$ superimposed onto $P_1$. **g** The ground truth ΔT superimposed onto $P_1$

Table 3 highlights the performance of the baseline methods, with a considerably low $F_1$ score ($F_1 = 0.11$ and 0.11) and IoU (IoU=0.07 & 0.07) for the best-performing threshold (γ=0.3) and *z*-score, respectively. This is paired with low precision ($\text {precision} = 0.10$ and 0.010) and moderate recall ($\text {recall} = 0.24$ and 0.26). This imbalance appears to be constant for all methods in Table 3, besides our highest fixed threshold (γ=0.5), where the model seems more conservative. Overall, most methods appear to have a significantly higher recall, indicating a tendency to identify more potential changes at the expense of precision. This systematic overestimation by *z*-score can be seen in Fig. 4.

This overestimation of the tumour region is demonstrated not only in our *z*-score results, as the fixed threshold appears to, for γ=0.5, filter out more noisy changes than the z-score approach, but at the cost of potentially significant changes. This, at times, results in problematic decisions as these change maps are purely filtered on intensity values and therefore filter out significant and insignificant changes based on the threshold technique. Figure 5 c and d illustrate this trade-off between sensitivity and specificity. Further results will be using γ=0.3 for our fixed threshold prediction.

### RiA

The registration-invariant approach (RiA) is trained and tested on different combinations of augmented data. We first verify the best model configuration on only shifted images and use this information to perform more augmentations (rotations) for the sake of imitating unregistered pairs. We set the fixed threshold to γ=0.3 for evaluation purposes. Moreover, augmentations are applied to baseline methods post-difference map generation when comparing them to our RiA, to generate a visually appealing comparison.

To determine the optimal hyperparameters for our loss function, we conducted an exhaustive grid search, evaluating all possible combinations of margin m and threshold τ. Table 4 shows a strong dependence on the margin parameter, with models trained without a margin (m=0.0) exhibiting significantly lower $F_1$ scores ($F_1 = 0.03$).

**Fig. 6:**
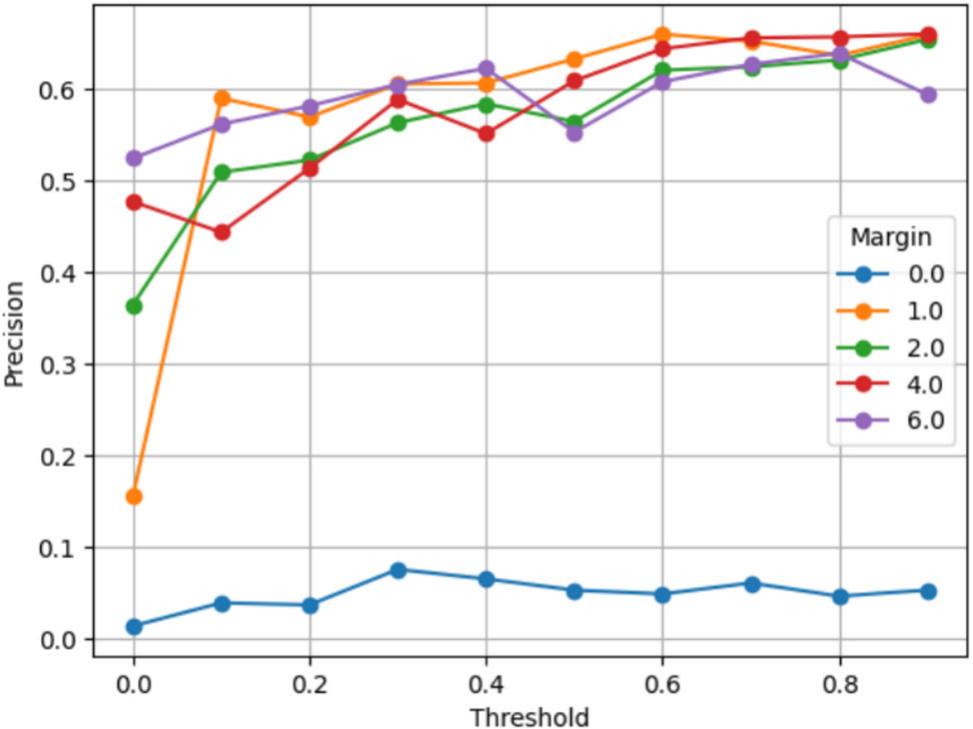
Precision of the RiA for different margin and threshold configurations

The highest $F_1$ score was achieved at m=4.0 and τ=0.7, with similar performance observed in configurations where m≥1.0 and $\tau \ge 0.4$. A slight reduction in $F_1$ and IoU scores was noted when increasing the threshold to τ=0.9, though this configuration resulted in a higher recall (see Table 4). Additionally, a correlation is observed between the threshold τ and precision, as shown in Fig. 6. Across all margin values, precision steadily increases with increasing τ, reaching peak values within the range $0.3 \le \tau \le 0.9$.

Based on the results of the grid search, we proceed with the best-performing model configuration (m=4,τ=0.7). We explore the effect of additional augmentations on model performance, assessing whether these transformations improve the model’s ability to generalise and detect changes more reliably.

### Global Change Identification

To assess change between image pairs, we compute relative feature maps and derive a scalar distance measure that quantifies their overall dissimilarity. This distance measure serves as a decision criterion for distinguishing between changed and unchanged pairs. By comparing the computed distances to the assigned binary ground truth labels (y), we evaluate the effectiveness of our method in identifying changes.

**Fig. 7:**
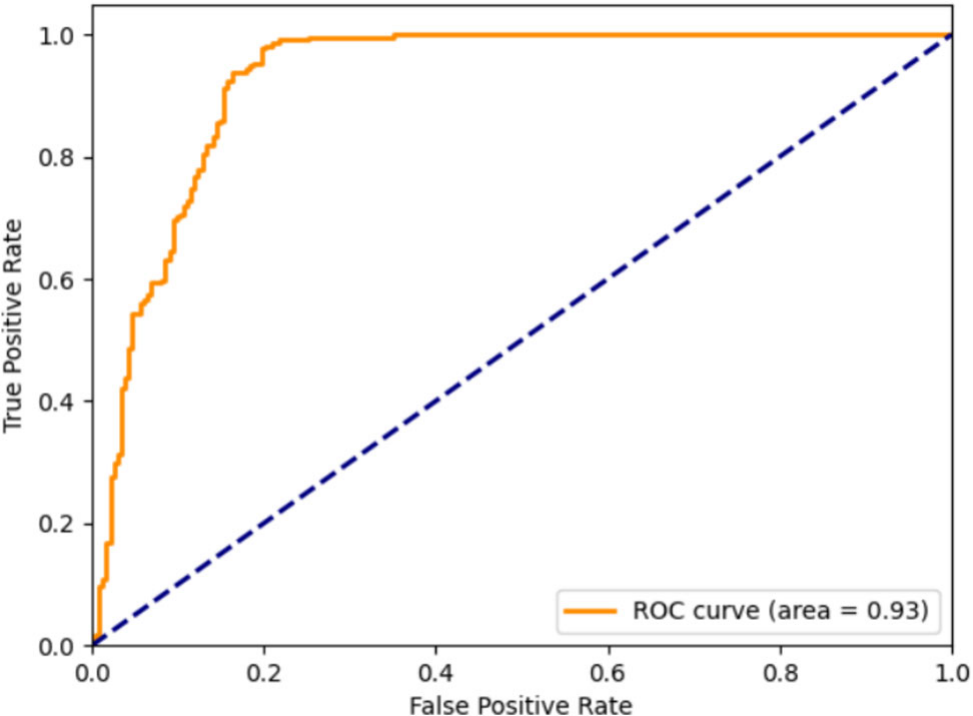
ROC curve for the RiA, showcasing the TPR against the FPR with the AUC for overall difference identification

A receiver operating characteristic (ROC) curve is generated, as shown in Fig. 7, to illustrate model performance across varying threshold values. The curve presents the trade-off between the true positive rate (TPR) and the false positive rate (FPR) as the decision boundary is adjusted.

The area under the curve (AUC = 0.93) indicates strong discriminatory ability between changed and unchanged image pairs. This performance is significantly higher than the random chance level (AUC = 0.50), suggesting that the model consistently assigns higher distance values to genuinely changed regions while maintaining low false positive rates. The ROC curve further highlights the consistency of the model’s performance across different threshold (γ) values.

**Table 4:** Performance summary of the RiA

Margin	Threshold	$F_1$ score$^{1}$	$IoU^{1}$	Precision$^{1}$	Recall$^{1}$
0.0	0.0	0.03	0.01	0.01	**0**.**92**
1.0	0.0	0.16	0.12	0.16	0.82
1.0	0.4	0.61	0.50	0.61	0.74
1.0	0.9	0.63	0.52	0.66	0.73
4.0	0.0	0.50	0.41	0.48	0.73
4.0	0.4	0.58	0.48	0.55	0.77
4.0	0.7	**0**.**68**	**0**.**57**	0.66	**0**.**80**
4.0	0.9	0.68	0.57	**0**.**66**	0.78
6.0	0.0	0.56	0.45	0.52	0.76
6.0	0.4	0.65	0.53	0.62	0.75
6.0	0.9	0.63	0.52	0.59	0.76

Performance metrics on shifted (between -50 and 50 pixels in each axis) data. The best-performing configurations for each metric are highlighted in bold

$^{1}$Decision thresholds used for all metrics are based on the optimal $F_1$ score

### Pixel-Level Localisation

Based on the results of the grid search, we proceed with the optimal model m=4,τ=0.7, as it demonstrated the most promising performance. For additional augmentations, we consider the previously mentioned optimal range of m≥1.0 and $\tau \ge 0.4$. As we are addressing the localisation of change maps, we proceed with our evaluation of changed pairs only (y=0).

Our RiA, trained on shifted image pairs, performs significantly better compared to the baseline methods. On metrics such as $$F_1$$ ($$F_1 = 0.68$$) and IoU (IoU=0.57), our model achieves a better score than our best baseline method ($$ F_1 = 0.11$$ and IoU=0.07) does on registered image pairs. A higher $$F_1$$ score indicates an improved balance between precision and recall, meaning that the model correctly identifies more changes while reducing false detections. Similarly, a higher IoU suggests a greater overlap between detected changes and the ground truth, demonstrating better localisation accuracy.

While $F_1$ and IoU provide a measure of overall performance, they do not explicitly differentiate between sensitivity and specificity. To further analyse the model’s performance, we observe that both precision (precision =0.66) and recall (recall =0.80) are higher than our best baseline result (precision =0.10 & recall =0.65).

**Fig. 8:**
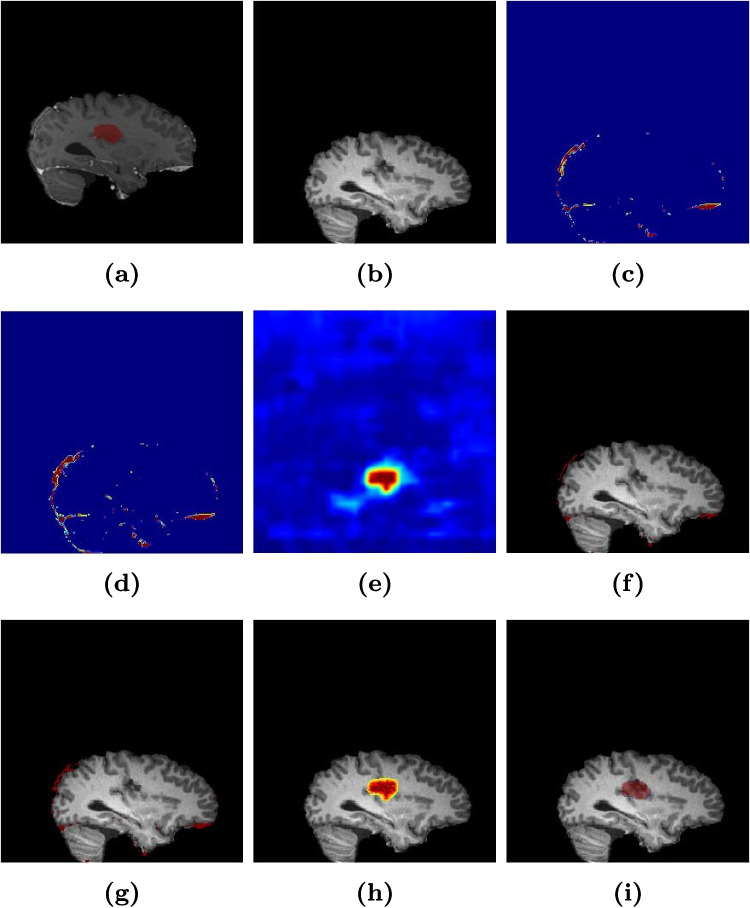
Sample results of ReMIND patient 39, showing a shifted sagittal mid-point slice. This patient’s preoperative and intraoperative states differ in post-processing due to the intraoperative being contrasted. **a** $T_0$ superimposed on $P_0$. **b** $T_1$ superimposed onto $P_1$. **c** Fixed threshold change map $\Delta \hat{T}_{thresh}$ for γ=0.3. **d** *Z*-score change map $\Delta \hat{T}_{z\text {-score}}$. **e** RiA ($\Delta \hat{T}^3_{model}$) prediction. **f** $\Delta \hat{T}_{thresh}$ superimposed onto $P_1$. **g** $\Delta \hat{T}_{z\text {-score}}$ superimposed onto $P_1$. **h** Sharpened RiA prediction superimposed onto $P_1$. **i** The ground truth ΔT superimposed onto $P_1$

This improvement can be seen in Fig. 8, where the model is able to correctly segment the predicted change map in a slightly shifted example ($F_1 = 0.85$ and IoU=0.74). This example pair also consisted of a contrasted intraoperative MRI scan (as opposed to a regular, non-contrasted preoperative scan), where it was still able to segment the right tumour region, despite the mismatch in the overall intensity distribution of certain tissue. The fixed threshold and *z*-score methods were unable to segment the right tumour region ($F_1 = 0.00$ and IoU =0.00).

**Fig. 9:**
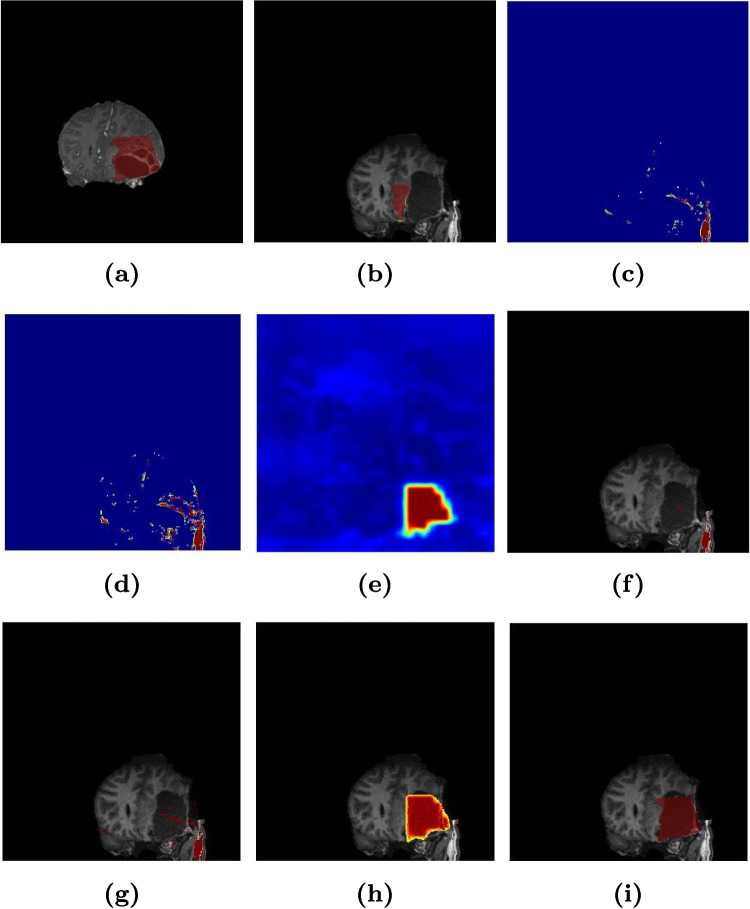
Sample results of ReMIND patient 25, showing an incorrectly extracted (using BET), shifted coronal slice. **a** $T_0$ superimposed on $P_0$. **b** $T_1$ superimposed onto $P_1$. **c** Fixed threshold change map $\Delta \hat{T}_{thresh}$ for γ=0.3. **d** *Z*-score change map $\Delta \hat{T}_{z\text {-score}}$. **e** RiA ($\Delta \hat{T}^3_{model}$) prediction. **f** $\Delta \hat{T}_{thresh}$ superimposed onto $P_1$. **g** $\Delta \hat{T}_{z\text {-score}}$ superimposed onto $P_1$. **h** Sharpened RiA prediction superimposed onto $P_1$. **i** The ground truth ΔT superimposed onto $P_1$

This invariance for shifted image pairs extrapolates to noise, which can be attributed to low-quality brain extraction or pre-processing. Figure 9 highlights an example of extreme noise in the form of skull retention. Our approach appears to still be capable of segmenting the right area, leaving out the residual tumour area as designed.

**Fig. 10:**
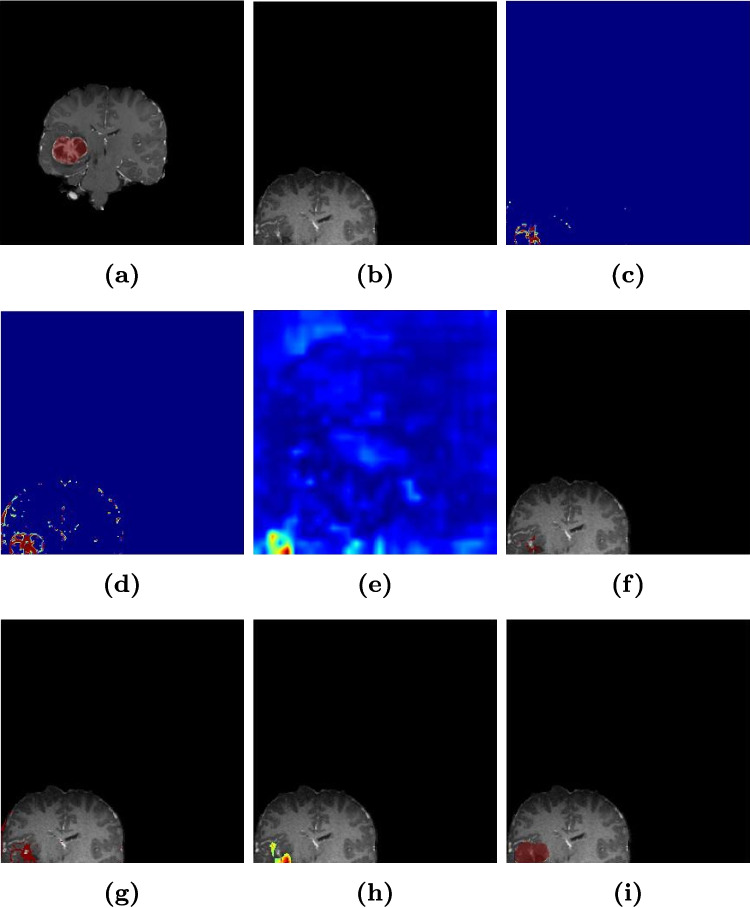
Sample results of ReMIND patient 57, showing a clipped, shifted coronal slice. **a** $T_0$ superimposed on $P_0$. **b** $T_1$ superimposed onto $P_1$. **c** Fixed threshold change map $\Delta \hat{T}_{thresh}$ for γ=0.3. **d** *Z*-score change map $\Delta \hat{T}_{z\text {-score}}$. **e** RiA ($\Delta \hat{T}^3_{model}$) prediction. **f** $\Delta \hat{T}_{thresh}$ superimposed onto $P_1$. **g** $\Delta \hat{T}_{z\text {-score}}$ superimposed onto $P_1$. **h** Sharpened RiA prediction superimposed onto $P_1$. **i** The ground truth ΔT superimposed onto $P_1$

Our approach also appears to perform relatively well ($F_1 = 0.77$ & IoU =0.62), in comparison to the baseline methods ($F_1 = 0.41$ and IoU=0.26 and $F_1 = 0.54$ and IoU =0.37), when applied to image pairs that are clipped due to shifting. Figure 10 shows an intraoperative state in which brain tissue is partially occluded by the image boundary.

### Additional Augmentation

**Fig. 11:**
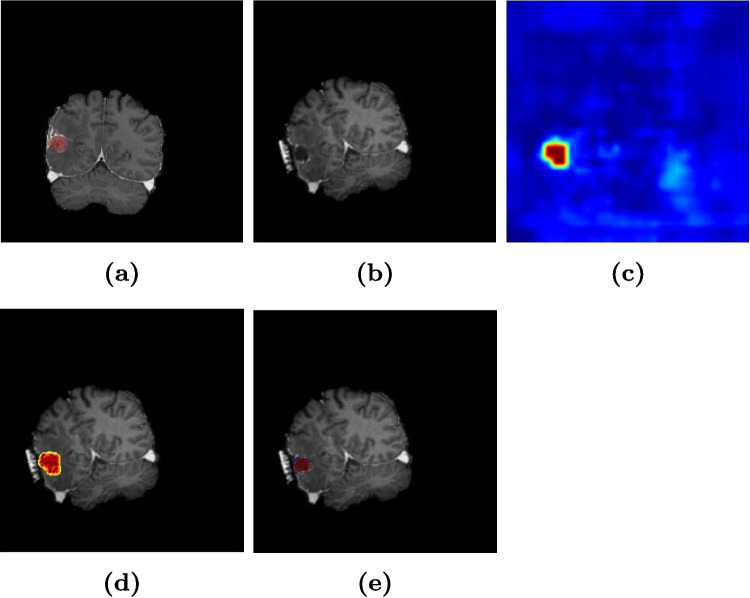
Sample RiA prediction result of ReMIND patient 15, showing a fully augmented coronal slice. **a** $T_0$ superimposed on $P_0$. **b** $T_1$ superimposed onto $P_1$. **c** RiA ($\Delta \hat{T}^3_{model}$) prediction. **d** Sharpened RiA prediction superimposed onto $P_1$. **e** The ground truth ΔT superimposed onto $P_1$

Moreover, we explore the effect of additional rotational augmentation on model performance, assessing whether these transformations improve the model’s ability to generalise and detect changes more reliably. Using rotation by -30 to 30$^{\circ }$, in line with observations reported by Seibert et al. [28], shifting by -50 to 50 pixels and the inherent noise within the ReMIND dataset, attributed to differences in data modalities and pre-processing errors such as remaining skull artefacts, we are able to fully test the robustness of our approach (Fig. 11).

Table 5 highlights the performance of the RiA on fully augmented data. We observe a moderate decrease in performance compared to the non-rotated setting (see Table 4), with $F_1 = 0.55$ and 0.68 and IoU =0.45 and 0.57, respectively. Furthermore, precision appeared to drop significantly (precision =0.45 and 0.66), while recall only decreased slightly when adding rotation (recall =0.75 and 0.80), respectively. This indicates that the model is more susceptible to making false positive predictions.

### Structural Changes

**Fig. 12:**
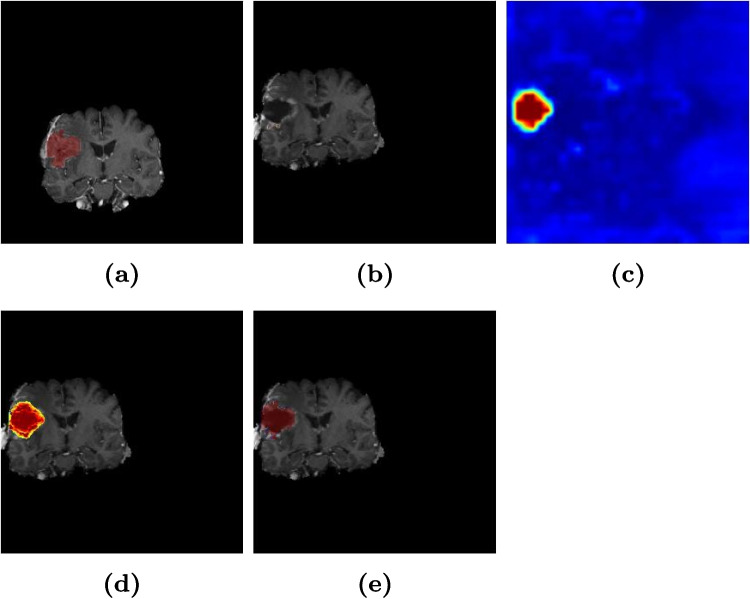
Sample panchromatic sharpening result of ReMIND patient 37, showing a fully augmented coronal slice. **a** $T_0$ superimposed on $P_0$. **b** $T_1$ superimposed onto $P_1$. **c** RiA ($\Delta \hat{T}^3_{model}$) prediction. **d** Sharpened RiA prediction superimposed onto $P_1$. **e** The ground truth ΔT superimposed onto $P_1$

Lastly, as can be seen in Fig. 12, our approach, when superimposed, applies panchromatic sharpening to our change map. This method applies the high-level features of the intraoperative and combines them with the intensity values of our change map. The sharpening in this image is capable of highlighting features in the intraoperative, adding contrast to high-intensity areas. In cases where resection results in heterogenous areas of tissue such as in Fig. 12, the differences are still clearly visible as opposed to the ground truth image with a regular overlay combined with a lower opacity (for transparency).

**Fig. 13:**
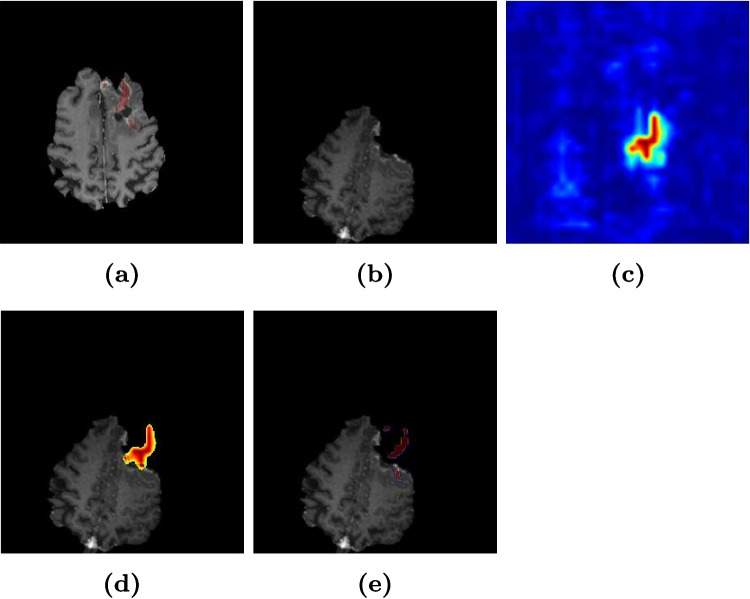
Sample panchromatic sharpening result of ReMIND patient 62, showing a fully augmented axial slice. **a** $T_0$ superimposed on $P_0$. **b** $T_1$ superimposed onto $P_1$. **c** RiA ($\Delta \hat{T}^3_{model}$) prediction. **d** Sharpened RiA prediction superimposed onto $P_1$. **e** The ground truth ΔT superimposed onto $P_1$

Panchromatic sharpening is able to contrast these high-intensity and low-intensity areas very well, but as can be seen in Fig. 13, the panchromatic sharpening is heavily dependent on the underlying tissue. The method performs well in regions with distinct structural features but fails in featureless or homogeneous areas. When the underlying tissue, due to brain tissue resection for example, is not available for sharpening, we are left with the regular feature map.

## Discussion

While our results demonstrate that the proposed approach generally outperforms simple DM-based baseline methods, a careful interpretation is necessary. The baseline methods, while simple, exhibit a consistent trade-off between recall and precision: low-threshold settings (e.g. γ=0.1,0.3) achieve high recall but low precision, whereas high thresholds (γ=0.5) improve precision at the cost of recall. These imbalances underscore the limitations of intensity-based methods in accurately segmenting tumour regions.

RiA overcomes many of these issues through contrastive learning and deep feature extraction, outperforming baseline methods on both global and pixel-level evaluations. The model demonstrates high AUC (AUC =0.93), indicating that the model produces discriminative feature pairs even in unregistered settings. Despite the lack of registration, RiA achieves a favourable balance between precision (precision =0.63) and recall (recall =0.79), indicating it captures subtle tumour changes while reducing false positives. The model is further shown to be robust to augmentations such as shifting, noise, and clipping, although performance drops with unoptimised rotation augmentations, pointing to the need for further hyperparameter tuning.

**Fig. 14:**
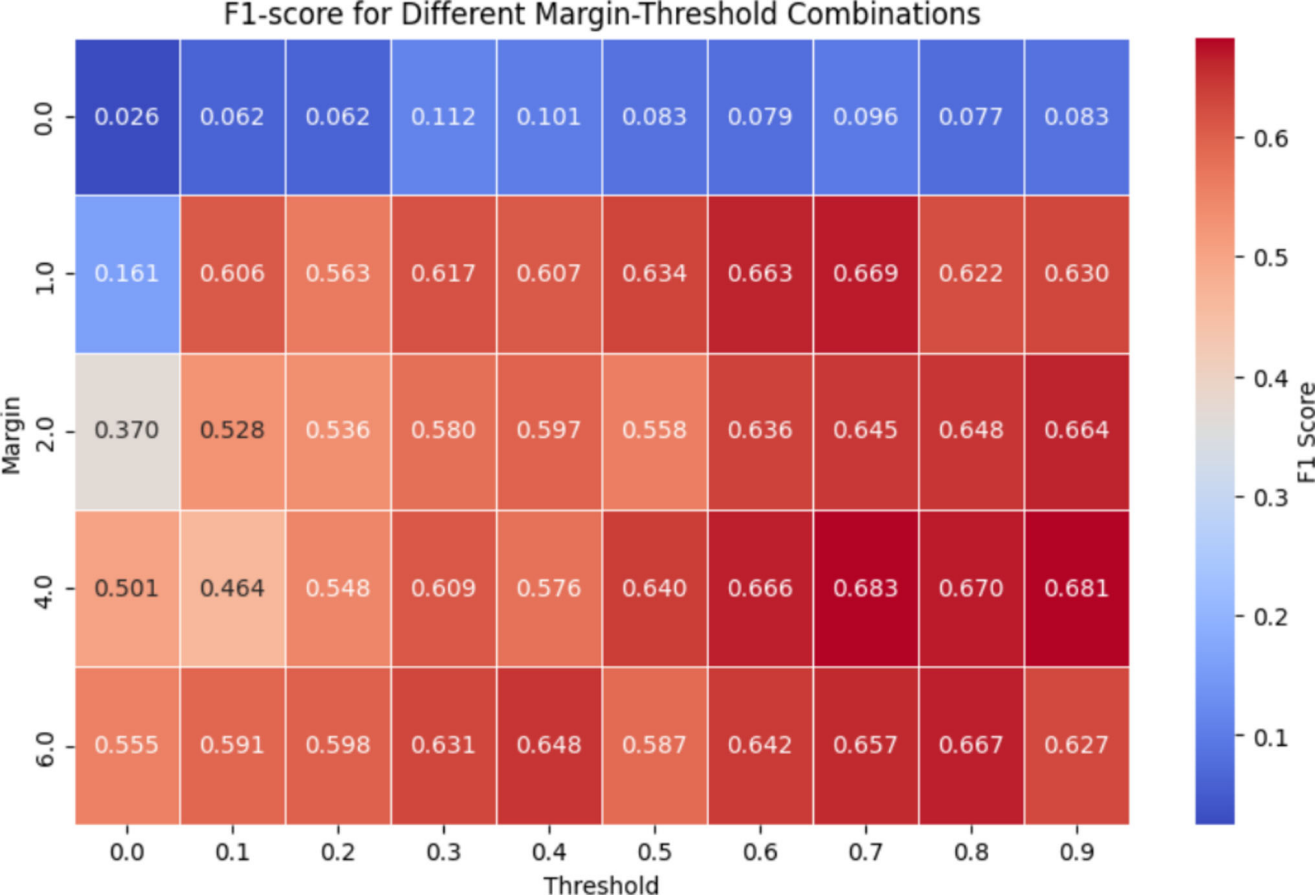
Grid search performed on the RiA, evaluating different margin and threshold configurations. The optimal $F_1$ scores were identified in the validation phase, with follow-up images shifted between -50 and 50 pixels horizontally and vertically

A grid search on the margin (*m*) and threshold (τ) hyperparameters, as shown in Fig. 14, confirms their critical role. Low margins (m=0) lead to poor feature separation, while the presence of a threshold (τ) reduces noise but may under-segment subtle changes. A complete absence of a threshold (τ=0) within TCL corresponds to the standard contrastive loss function, which consistently underperforms compared to configurations where a threshold is introduced. In other words, incorporating a threshold leads to more accurate results across all tested margin values. The optimal region was found at m≥1.0 and $\tau \ge 0.4$, balancing sensitivity and specificity. However, we acknowledge that a configuration is context-dependent.

Clinically, a recall of 0.79 indicates that RiA captures the majority of resected tumour pixels, while a precision of 0.63 shows that the predicted changes largely correspond to true resection, with limited false positives. Literature on glioma resection shows that even small amounts of residual tumour can affect overall survival [3]. While RiA does not detect every single resected pixel, many missed pixels are at the tumour margins, which are less likely to be clinically consequential, particularly when postoperative follow-up therapy is applied. Furthermore, the median preoperative tumour volume in the cohort was $17.83~\mathrm {cm^3}$, with an interquartile range of 6.31–35.87 cm$^3$, reflecting the variability in tumour sizes among patients [16]. Based on the observed precision of 0.63, approximately 37% of the pixels identified as resected may not correspond to actual tumour removal. For a tumour of 17.83 cm$^3$, this could translate to an overestimation of the resected volume by roughly 6–7 cm$^3$, which would remain as residual tumour. For larger tumours at the upper end of the interquartile range, the volume of potentially unrecognised residual tissue could exceed 13 cm$^3$. These values exceed the 5 cm$^3$ residual volume threshold previously reported to significantly affect survival and recurrence in glioblastoma [3], indicating that careful interpretation of predicted resection extent is critical.

Conversely, if the method is applied for change detection in growing tumours as opposed to tumour resection, recall becomes the primary metric of interest. Recall would reflect the fraction of newly grown tumour tissue successfully identified. In this scenario, a recall of 0.79 would indicate that RiA detects most of the tumour growth, with the remaining 21% of growth representing potentially undetected tumour growth. This demonstrates that RiA can provide meaningful quantitative information both for assessing resection and for monitoring tumour progression, depending on the clinical application.

One of the primary limitations of our approach is the effective dataset size. Although our dataset contains over 5000 image pairs, the number of unique patients is significantly lower (*n* = 65). As a result, the model may inadvertently learn associations between multiple slices from the same patient, leading to overfitting. This is a well-documented issue in segmentation models [23], particularly when splitting occurs at the slice level rather than at the patient level. A sensible way to mitigate this issue would be to split the data at the patient level (excluding certain patients from training) or to apply extensive data augmentation. However, we argue that this would not fully resolve the underlying issue, as an additional problem lies in the data quality, which can be attributed to the long pre-processing pipeline such as manual slice creation and naive tumour mask subtraction. Further data quality issues arise due to relaxation in accepted data points as MRI patient data is scarce (e.g. usage of different acquisition parameters for a singular image pair such as T1-weighted and T2-weighted). These data quality issues reduce generalisability and introduce illogical pairs, skewing the training process. One explanation for illogical pairs, such as tumours that increase in size post-resection, is due to the matching of different image modalities. This, at times, can mistakenly match slices of different spatial positions between tumour segmentations and brain scans. Other, less frequent cases may affect generalisability, such as the low representation of preoperative swelling or fluid accumulation. These cases, often marked by hypointense regions in the tumour area, introduce variations that are not consistently captured across patients, making them harder for the model to generalise to. Improving dataset consistency and quality, along with better augmentation strategies, is necessary. Furthermore, panchromatic sharpening used for visualising structural changes performs well when change regions are contrasted, but struggles in homogeneous regions.

**Table 5:** Performance summary of the RiA on fully augmented data

Margin	Threshold	$F_1$ score$^{1}$	$IoU^{1}$	Precision$^{1}$	Recall$^{1}$
4.0	0.7	0.55	0.45	0.45	0.75

Performance metrics on shifted (between -50 and 50 pixels in each axis) data. The best-performing configurations for each metric are highlighted in bold

$^{1}$Decision thresholds used for all metrics are based on the optimal $F_1$ score

Future research should investigate the feasibility of volumetric approaches. Integrating 3D segmentation models within the Siamese network together with volumetric data would bypass a vast majority of the pre-processing requirements such as labelling and slice generation (which requires matching each preoperative slice with its corresponding intraoperative slice). Moreover, we propose the integration of MRI scans acquired at later postoperative timepoints, rather than intraoperative images. This shift would enable the detection of longer-term neuroplastic changes such as axonal sprouting, which can strengthen previously damaged pathways in the brain [17]. This use case would primarily justify the usage of panchromatic sharpening as it allows for the changed area to decompress after tumour resection, resulting in re-emerging anatomical features in the changed area of the postoperative. Lastly, we suggest the implementation of our concept to a broader range of medical fields, disease progression being one of them.

## Conclusion

We proposed a registration-invariant approach to change detection which was able to leverage the features extracted from a segmentation model used in a Siamese network and construct a change map. In comparison to a subtraction baseline approach, our method was able to successfully outperform the baseline in the identification of changes and localisation of pixel-level changes whilst effectively highlighting structural modifications. Consequently, it provides a strong basis for change detection in the form of disease progression in the medical field. Beyond a continued optimization of this approach in the future, the incorporation of a postoperative to fully exploit the panchromatic sharpening should be explored. Furthermore, the application of our concept to 3D data would be the logical next step in this research.

## Data Availability

The REMIND dataset is available at https://www.cancerimagingarchive.net/collection/remind/.

## Code Availability

Full code used for this study will be available at https://github.com/adiljahouh/Thesis-Change-Detection-in-MRI.
